# Beyond neurofilaments: a multidimensional blood signature for amyotrophic lateral sclerosis

**DOI:** 10.1093/braincomms/fcag231

**Published:** 2026-06-17

**Authors:** Eleanor V Thomas, Devesh C Pant

**Affiliations:** Department of Neurology, Emory University School of Medicine, Atlanta, GA 30322, USA; Department of Cell Biology, Emory University School of Medicine, Atlanta, GA 30322, USA

## Abstract

This scientific commentary refers to ‘Blood-based biomarker discovery in motor neuron disease using nucleic acid-linked immuno-sandwich assay’, by Bozkurt *et al*. (https://doi.org/10.1093/braincomms/fcag180).

This scientific commentary refers to ‘Blood-based biomarker discovery in motor neuron disease using nucleic acid-linked immuno-sandwich assay’, by Bozkurt *et al*. (https://doi.org/10.1093/braincomms/fcag180).

Motor neuron disease (MND), including amyotrophic lateral sclerosis (ALS), remains one of the most devastating neurodegenerative disorders, marked by clinical heterogeneity, delayed diagnosis, and limited therapeutic success. A central barrier to progress has been the lack of accessible, reliable targets that capture disease biology while enabling early diagnosis and monitoring. New precision proteomic technologies have transformed our ability to study ALS/MND through accessible patient biospecimens such as blood and CSF. Highly sensitive, multiplexed proteomic platforms, including Nucleic Acid Linked Immuno-Sandwich Assay (NULISA), Olink, SomaScan, and Simoa have enabled increasingly detailed molecular profiling of ALS patient biofluids and accelerated the discovery of blood-based biomarkers associated with disease diagnosis, progression, and biological heterogeneity.

## Expanding the reach of blood-based biomarkers

Historically, biomarker discovery in ALS has relied heavily on CSF. Blood-based biomarkers offer a practical alternative but have suffered from insufficient sensitivity. In their recent article in *Brain Communications*, Bozkurt *et al*.^1^ apply the NULISAseq 120-plex CNS Disease Panel alongside Simoa Neurology 2-Plex validation to interrogate serum samples from 48 individuals with MND in the Scottish Motor Neuron Disease Register (CARE-MND) and 38 controls. Importantly, neurofilament light chain (NfL) remains the top performing biomarker, with high diagnostic accuracy (AUC ∼ 0.92). The study moves beyond validation to uncover a broader proteomic signature that may better capture disease heterogeneity by identifying 20 significantly altered proteins, including NfL, NfH, pTau-181, pTau-217, pTau-231, total tau, FABP3, Aβ38, and Aβ40. This study provides important independent confirmation of prior ALS biomarker studies by us and others and strengthens the growing evidence that elevation of phosphorylated tau and amyloid-beta species represents a stable and reproducible blood-based signature of ALS.^2-5^

## Tau beyond Alzheimer’s disease: a marker of motor neuron degeneration?

Particularly notable is the replication of increased pTau-181, pTau-217, and pTau-231, findings that align closely with recent plasma proteomics studies and reinforce the conclusion that tau dysregulation is a consistent feature of ALS biology rather than a signal specific to Alzheimer disease (AD). Traditionally associated with AD, tau pathology has not been considered central to ALS/MND. Yet growing evidence suggests that tau dysregulation in ALS may reflect broader neurodegenerative processes, including degeneration of the motor system. This study reinforces emerging reports of elevated plasma pTau-181 in ALS and extends them by demonstrating concordant increases across multiple tau epitopes.^6^ The authors propose an intriguing hypothesis: that serum tau may originate, at least in part, from peripheral sources such as spinal motor neurons, rather than solely from CNS pathology. This idea is supported by the presence of high molecular weight (HMW) ‘big tau’ isoforms in neurons with long peripheral projections and by studies showing that a low molecular weight (LMW)-specific plasma pTau assay shows greater elevation in AD than ALS, while non-specific pTau assays measuring both HMW and LMW pTau show elevation in ALS and AD.^7^ This shifts the conceptual framework of tau from a purely CNS biomarker to one that may reflect lower motor neuron degeneration more directly. It also raises the possibility that tau could serve as a pharmacodynamics marker in clinical trials, complementing neurofilaments. However, caution is warranted. Tau biology is complex, and its elevation may reflect overlapping neurodegenerative pathways rather than disease-specific mechanisms.

## Peripheral signals: FABP3 and the muscle hypothesis

An especially intriguing finding is the elevation of FABP3, which complements recent work implicating skeletal muscle and peripheral metabolic dysfunction in ALS pathobiology.^8,9^ As a muscle-enriched fatty acid transport protein that has been linked to neuronal injury and muscle pathology,^10^ FABP3 provides a biologically plausible link between circulating proteomic changes and the muscle injury, mitochondrial dysfunction, and bioenergetic stress increasingly recognized in ALS. Its correlation with phosphorylated tau raises the possibility that muscle metabolic dysfunction and neurodegeneration are biologically coupled processes in ALS, rather than independent downstream consequences of motor neuron loss. The authors speculate that FABP3 may reflect peripheral tissue involvement, including skeletal muscle dysfunction, a hypothesis that resonates with emerging models of ALS as a multisystem disease rather than a neuron-centric disorder. For the field, this is a subtle but important shift. Biomarkers like FABP3 may not only indicate neurodegeneration but also capture metabolic and peripheral stress responses, offering a systems level view of disease biology.

## Amyloid and co-pathology: signal or noise?

The study also reports elevated amyloid-beta peptides (Aβ38, Aβ40) in ALS serum. While amyloid is a hallmark of AD, its role in MND remains unclear. Bozkurt *et al*.^1^ appropriately interpret these findings cautiously, suggesting that amyloid markers may serve as supplementary biomarkers rather than primary drivers of disease.^1^ Their inclusion in the biomarker panel, however, underscores the complexity of neurodegeneration, where molecular signatures often overlap across disease boundaries. This raises a broader question: should biomarker development prioritize disease specificity or embrace shared neurodegenerative pathways? Multiplexed approaches like NULISA may allow both identifying core signatures while also revealing disease-specific subtypes. As with many early biomarker studies, several limitations should be acknowledged. This is a single-centre study with a modest sample size, and larger multi-centre validation studies will be necessary to determine the generalizability and clinical utility of these findings. Future efforts integrating large, multi-centre, multi-omics datasets will be especially valuable for defining biomarker signatures associated with clinical heterogeneity, disease progression, and therapeutic response. This study adds important support to a rapidly evolving field and further reinforces the view that blood-based multiplex proteomics may help identify useful markers of disease that will someday be applied to improve ALS research and clinical care.

## Conclusion

**Figure 1:**
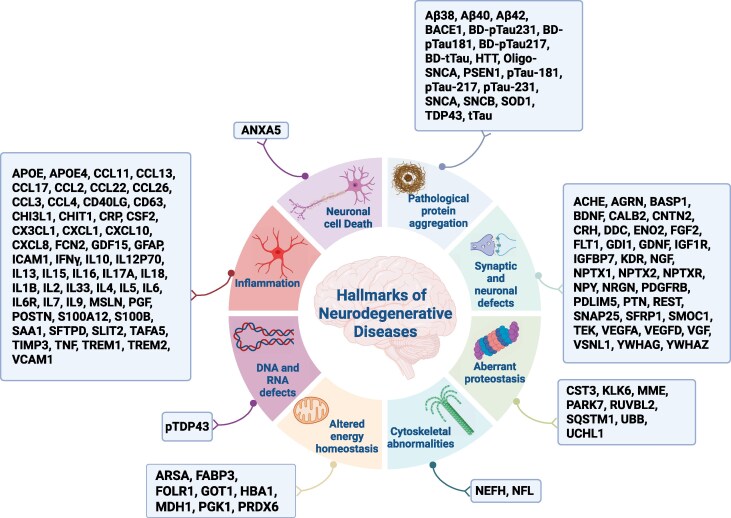
**The CNS 120 panel captures convergent molecular hallmarks of neurodegeneration.** This schematic diagram illustrates the principal biological pathways represented within the NULISA CNS 120 biomarker panel. Representative proteins associated with each pathway are shown in the surrounding panels. By enabling simultaneous measurement of diverse yet interconnected molecular processes, the CNS 120 panel provides an integrated framework for biomarker discovery, mechanistic insight, and longitudinal monitoring of neurodegenerative disease progression. Created in BioRender. P, D. (2026) https://BioRender.com/jqt180g.

Bozkurt *et al*.^1^ provide a compelling demonstration of how ultra-sensitive proteomic technologies can reshape biomarker discovery in ALS/MND.^1^ The broader significance of this work lies in its support for a more integrated model of ALS biomarker biology, rather than a purely neuron-restricted neurodegenerative disease. By validating established markers and uncovering new candidates, their study advances both the technical and biological understanding of the disease. Perhaps most importantly, the work challenges a narrow, neuron-centric view of ALS. The emerging biomarker profile, encompassing neurofilaments, tau, metabolic proteins, and amyloid, suggests a complex, multisystem disorder with contributions from both central and peripheral tissues (Fig. 1). The road to clinical translation remains long, requiring larger cohorts, longitudinal validation, and mechanistic studies. Yet this study marks a meaningful step toward a future where a simple blood test can inform diagnosis, monitor progression, and guide therapy in ALS.

## Data Availability

Data sharing is not applicable to this article as no new data were created or analysed.

## References

[1] Bozkurt H, Reid KR, Newton J, et al Blood-based biomarker discovery in motor neuron disease using nucleic acid-linked immuno-sandwich assay. Brain Commun. 2026:10.1093/braincomms/fcag180. PMC13312953 42375130

[2] Dulski J, Boddapati AK, Risi B, et al Targeted plasma proteomics uncover proteins associated with KIF5A-linked SPG10 and ALS spectrum disorders. HGG Adv. 2026;7(1):100498. 40873038 10.1016/j.xhgg.2025.100498 PMC12903090

[3] McEachin ZT, Chung M, Stratton SA, et al Molecular impact of antisense oligonucleotide therapy in C9orf72-associated ALS. Cell. 2025;188(23):6424–6435.e17. 40865525 10.1016/j.cell.2025.07.045 PMC13054592

[4] Steffke C, Baskar K, Bachhuber F, et al Targeted proteomics upon treatment with tofersen identifies novel response markers for superoxide dismutase 1-linked amyotrophic lateral sclerosis. Ann Neurol. 2025;98(6):1318–1334. 40781905 10.1002/ana.70025 PMC12682945

[5] Thomas EV, Han C, Kim WJ, et al ALS plasma biomarkers reveal neurofilament and pTau correlate with disease onset and progression. Ann Clin Transl Neurol. 2025;12(4):714–723. 39913612 10.1002/acn3.70001 PMC12040516

[6] Cousins KAQ, Shaw LM, Shellikeri S, et al Elevated plasma phosphorylated tau 181 in amyotrophic lateral sclerosis. Ann Neurol. 2022;92(5):807–818. 35877814 10.1002/ana.26462 PMC9588516

[7] Janelidze S, Ashton NJ, Orduna Dolado A, et al A comparison of p-tau assays for the specificity to detect tau changes in Alzheimer's disease. Alzheimers Dement. 2025;21(4):e70208. 40289884 10.1002/alz.70208 PMC12035549

[8] Chia R, Moaddel R, Kwan JY, et al A plasma proteomics-based candidate biomarker panel predictive of amyotrophic lateral sclerosis. Nat Med. 2025;31(10):3440–3450. 40830661 10.1038/s41591-025-03890-6 PMC12532604

[9] Dergai O, Wuu J, Koziczak-Holbro M, et al Skeletal muscle biomarkers of amyotrophic lateral sclerosis: A Large-Scale, Multi-Cohort Proteomic Study. Ann Neurol. 2026;99(2):393–407. 41020397 10.1002/ana.78046 PMC12894501

[10] Sepe FN, Chiasserini D, Parnetti L. Role of FABP3 as Biomarker in Alzheimer's disease and synucleinopathies. Future Neurol. 2018;13(4):199–207.

